# Next-Generation Probiotics and Chronic Diseases: A Review of Current Research and Future Directions

**DOI:** 10.1021/acs.jafc.4c08702

**Published:** 2024-11-26

**Authors:** Ashutosh Tiwari, Dyah Ika Krisnawati, Erna Susilowati, Chinmaya Mutalik, Tsung-Rong Kuo

**Affiliations:** † International Ph.D. Program in Biomedical Engineering, College of Biomedical Engineering, Taipei Medical University, Taipei 11031, Taiwan; ‡ Department of Nursing, Faculty of Nursing and Midwifery, Universitas Nahdlatul Ulama Surabaya, Surabaya, 60237 East Java, Indonesia; § Akademi Kesehatan Dharma Husada Kediri, Kediri, 64118 East Java, Indonesia; ∥ Graduate Institute of Nanomedicine and Medical Engineering, College of Biomedical Engineering, Taipei Medical University, Taipei 11031, Taiwan

**Keywords:** Next-Generation Probiotics, Chronic Diseases, Synthetic Biology, Microbiome-Based Therapies, Hydrocolloids

## Abstract

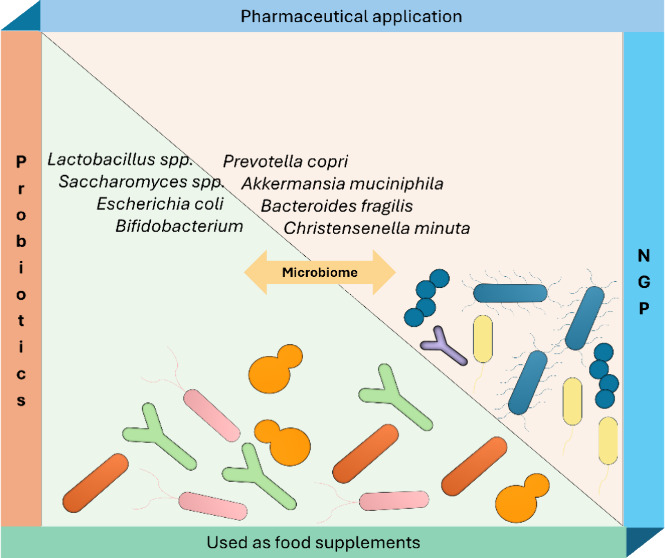

The burgeoning field of microbiome research has profoundly reshaped our comprehension of human health, particularly highlighting the potential of probiotics and fecal microbiota transplantation (FMT) as therapeutic interventions. While the benefits of traditional probiotics are well-recognized, the efficacy and mechanisms remain ambiguous, and FMT’s long-term effects are still being investigated. Recent advancements in high-throughput sequencing have identified gut microbes with significant health benefits, paving the way for next-generation probiotics (NGPs). These NGPs, engineered through synthetic biology and bioinformatics, are designed to address specific disease states with enhanced stability and viability. This review synthesizes current research on NGP stability, challenges in delivery, and their applications in preventing and treating chronic diseases such as diabetes, obesity, and cardiovascular diseases. We explore the physiological characteristics, safety profiles, and mechanisms of action of various NGP strains while also addressing the challenges and opportunities presented by their integration into clinical practice. The potential of NGPs to revolutionize microbiome-based therapies and improve clinical outcomes is immense, underscoring the need for further research to optimize their efficacy and ensure their safety.

## 1 Introduction

**Figure 1:**
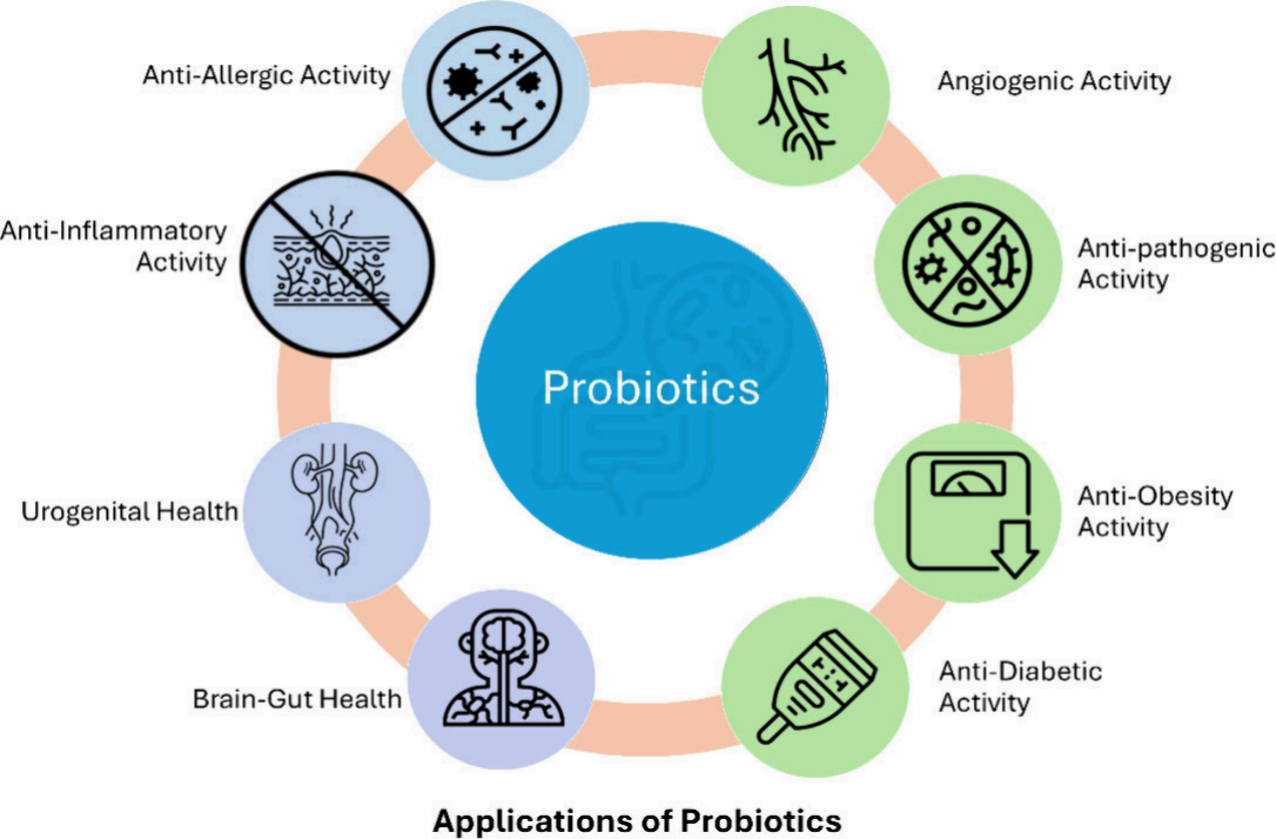
Applications of probiotics in health and disease prevention.

Microbiome research has transformed our understanding of human microbiology, allowing us to associate microflora with diseases and identify potential therapeutic solutions such as probiotics and fecal microbiota transplantation (FMT). Probiotics are live microorganisms that confer health benefits when consumed in adequate amounts.^1^ These microorganisms have a long history of use in traditional fermented foods, but it was not until the late 1960s that the term “probiotics” was coined by Nobel laureate Elie Metchnikoff.^2^ Today, probiotics have gained widespread recognition for their potential to improve human health through their effects on the gut microbiome (Figure 1).^3,4^

**Figure 2:**
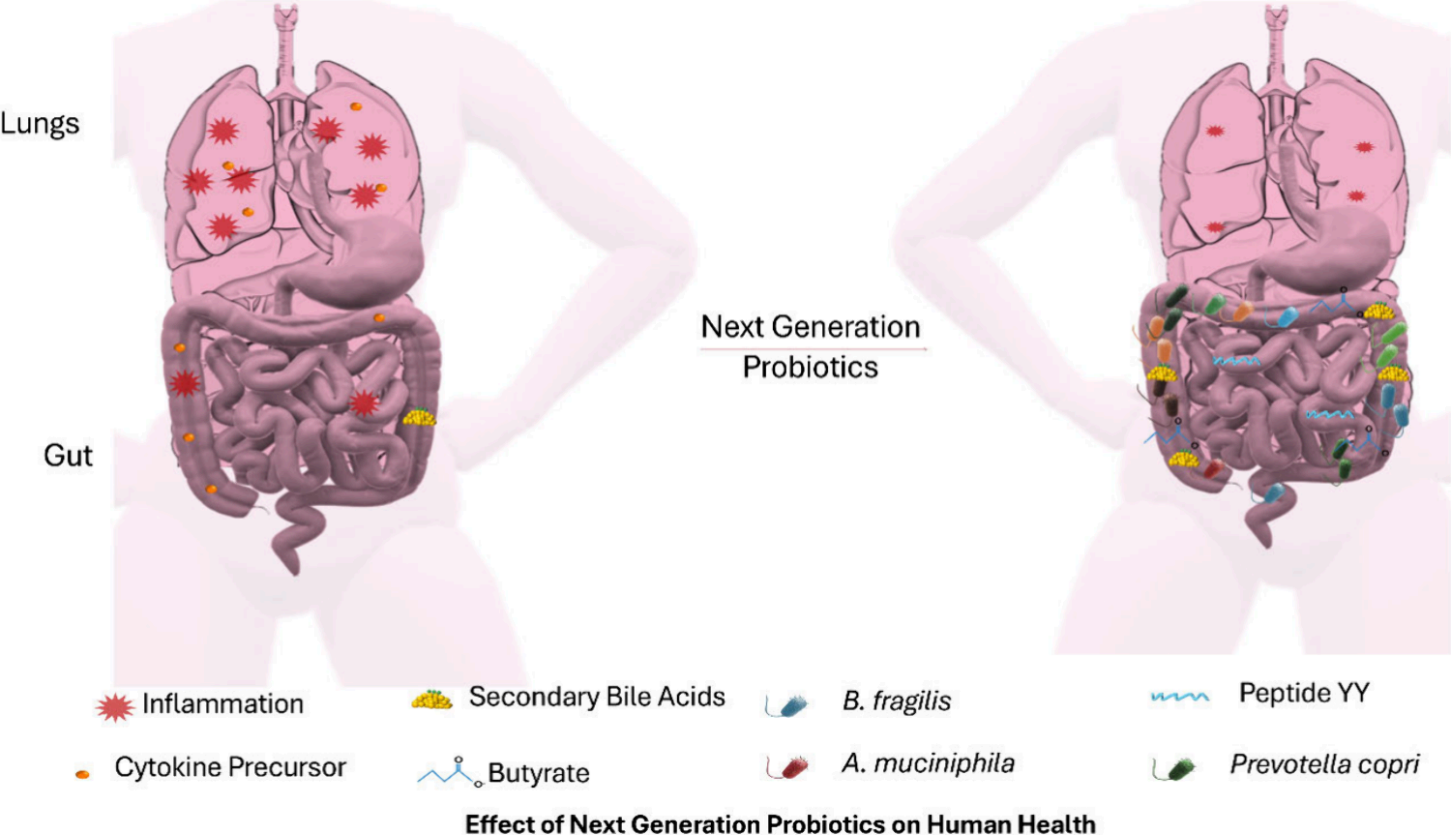
Impacts of probiotics on inflammation and gut health. The figure illustrates how probiotics can reduce inflammation in the lungs and gut by introducing beneficial bacteria and substances that promote a healthy immune response and gut environment.

Despite the potential benefits of probiotics, their clinical efficacy and mechanisms of action remain unclear. Moreover, many of the probiotic strains available on the market lack consistent and high-quality clinical evidence to support their use in preventing or treating specific diseases.^5,6^ Also, the effectiveness of probiotics may vary widely among individuals and disease states, which makes it challenging to identify specific strains for targeted interventions. Similarly, current probiotics might not survive the harsh conditions of the gastrointestinal (GI) tract (GIT), limiting their effectiveness.^7^ To overcome these limitations, there is a need for NGPs that target specific disease states and interact more effectively with the host microbiome. NGPs have the potential to be tailored to individual needs, based on the composition of the microbiome and the specific disease being treated.^8^ They can also be designed to survive GIT’s harsh conditions and have greater stability and viability. Eventually, advances in high-throughput sequencing and bioinformatics such as metagenomic sequencing to identify potential NGPs,^9^ functional annotation studies for metabolic pathway analysis,^10^ Genome-Wide Association Studies (GWAS) for host-microbe interaction,^11^ and transcriptome analysis^12^ enabled the identification of new microbial strains with potential health benefits that can serve as candidates for NGPs.^13^ Advances in high-throughput sequencing have identified gut microbes with health benefits^14^ that could serve as candidates for next-generation probiotics (NGPs) (Table 1). By addressing these limitations, NGPs have the potential to revolutionize the field of microbiome-based therapies and improve clinical outcomes for a wide range of chronic diseases. Examples of potential next-generation probiotics include: *Faecalibacterium prausnitzii, Akkermansia muciniphila, Bacteroides fragilis,* etc. These probiotic strains were studied for their effects on diseases and certain health conditions (Figure 2).^15^

**Table 1:** Comparisons of Fecal Microbiota Transplantation (FMT) vs Next-Generation Probiotics (NGPs)

aspect	fecal microbiota transplantation (FMT)	next-generation probiotics (NGPs)
definition	transplantation of stool from a healthy donor into the gastrointestinal tract of a patient to restore the balance of the gut microbiota^120^	engineered probiotics designed to target specific strains or metabolic pathways for therapeutic outcomes, often utilizing advanced technologies such as synthetic biology and gene editing^121^
mechanism of action	introduces a complex community of bacteria to reestablish a healthy gut microbiotic balance^122^	modulates the gut microbiota by introducing specific beneficial strains, producing targeted metabolites, or expressing therapeutic proteins^123^
applications	primarily used to treat recurrent *Clostridium difficile* infections, while being investigated for other conditions such as irritable bowel disease (IBD), irritable bowel syndrome (IBS), and metabolic syndrome^124^	targeted treatment for chronic diseases like diabetes, obesity, cardiovascular diseases, IBD, and potentially other conditions through specific bacterial strains and engineered functionalities^125^
efficacy	high efficacy in treating recurrent *C. difficile* infections; variable efficacies in other conditions due to the complexity and variability of donor microbiota^126^	potential for high efficacy against specific chronic conditions due to targeted action; requires more clinical trials to fully establish effectiveness across various conditions^37^
safety	generally considered safe, but risks include the transfer of pathogenic organisms and adverse reactions to donor stool^127,128^	generally considered safe with fewer side effects; potential risks related to long-term effects and interactions with the host microbiome^128^
regulatory status	increasingly accepted in clinical practice with specific guidelines for donor screening and stool processing; the Food and Drug Administration (FDA) classifies it as an investigational new drug for certain conditions.^129^	emerging field with regulatory frameworks still developing; classified under different regulatory categories depending on the specific engineering methods and therapeutic claims^130^
personalization	limited by the availability of suitable donors and variability in donor microbiotic compositions; difficult to customize for individual patients^131^	high potential for personalization based on individual microbiomic compositions and specific health needs; can be tailored using genomic and metagenomic data.^131^
cost and accessibility	can be costly due to the need for donor screening, stool processing, and clinical administration; accessibility may vary based on regulatory and healthcare infrastructure	potential for lower costs and broader accessibility once established; production can be standardized, and administration may be simpler compared to FMT
research and development	well-established for *C. difficile* infections with ongoing research for other conditions; research has focused on optimizing donor selection and understanding long-term effects.	rapidly growing field with significant research into developing new strains, understanding mechanisms of action, and conducting clinical trials to demonstrate efficacy and safety for various chronic conditions
long-term effects	long-term effects are still being studied; concerns about the stability of introduced microbiota and potential for unforeseen health impacts	long-term effects need further research; engineered strains designed for stability and targeted action potentially offer more-predictable outcomes

This review article provides an overview of the current state of probiotics research and focuses on NGPs’ potential for preventing and treating chronic diseases such as diabetes, obesity, and cardiovascular diseases (CVDs) while discussing effects of NGPs on other health conditions, exploring their physiological characteristics, safety, pathogenicity, drug resistance, and effects on host health, as well as challenges and opportunities for their use in clinical practice. These chronic diseases were strategically chosen because of their global relevance in health as diabetes, obesity and CVDs are among the most prevalent health conditions.^16^ Obesity and diabetes together contribute to unfavorable cardiovascular biomarkers and increased mortality risks.^17^ Research has established a strong connection between the gut microbiota and chronic diseases. Dysbiosis has been implicated in the development and progression of type 2 diabetes, CVDs and obesity.^18^ Probiotics have shown potential in lipid metabolism, which can lower cholesterol levels and reduce cardiovascular risk.^19^

## 2 Understanding the Current State of Probiotics Research

The current state of probiotics research is characterized by a rapidly expanding body of knowledge on the microbiome and its role in human health and disease.^20^ Despite some promising findings, there is still a lack of consistent and high-quality clinical evidence to support the use of probiotics in preventing or treating specific diseases. Furthermore, challenges such as a lack of standardization and quality control in probiotic production, and an incomplete understanding of the mechanisms through which probiotics interact with the host microbiome and immune system, limit their clinical effectiveness.^21^ To overcome these challenges, there is a growing interest in NGPs that can be tailored to individual needs which address these limitations. NGPs may hold great potential to improve clinical outcomes for a wide range of chronic diseases, by utilizing new microbial strains and addressing the limitations of current probiotics.

**Figure 3:**
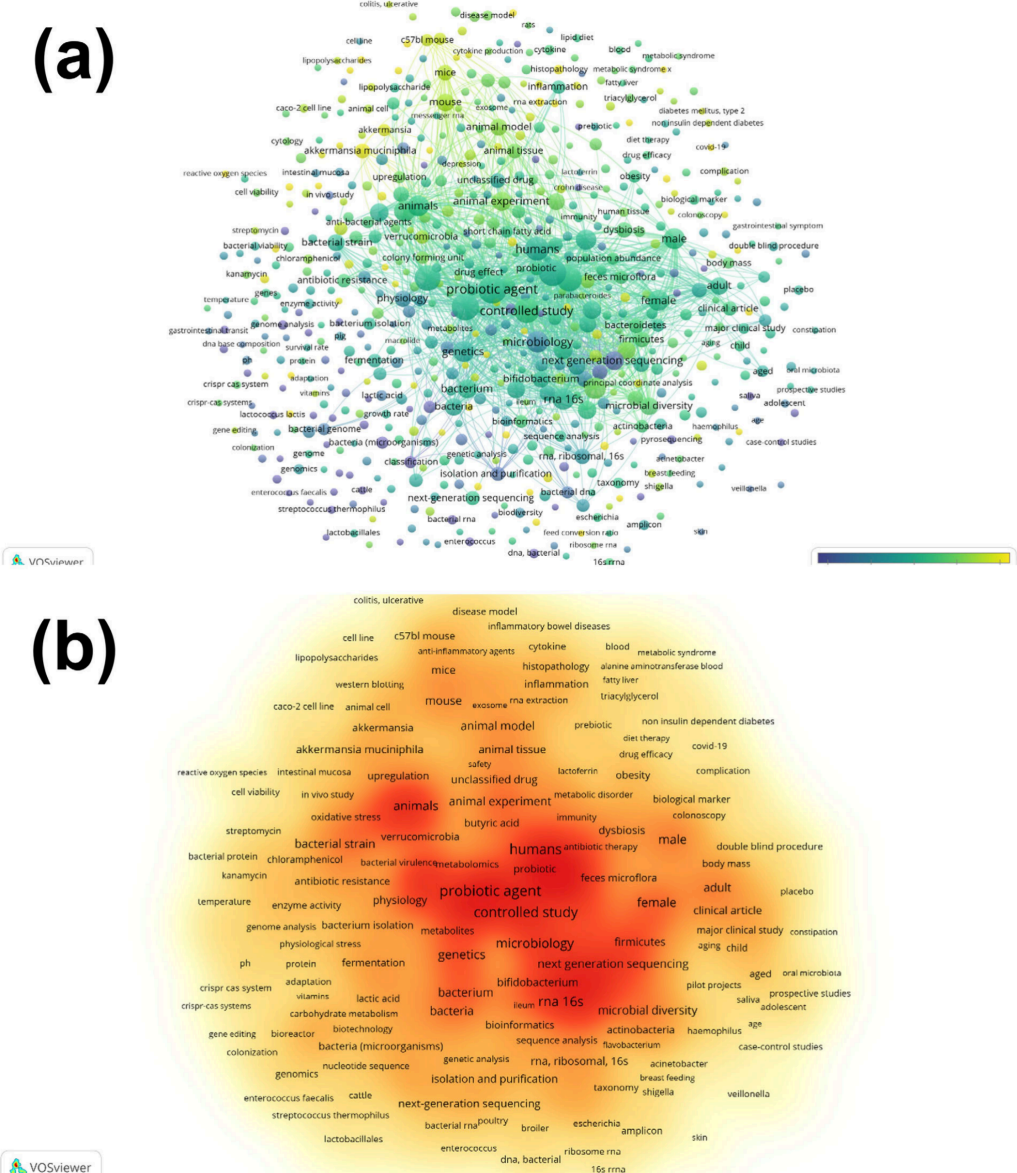
(a) Network visualization and (b) Density visualization of keywords related to probiotic interactions and their functions.^22^

Probiotics have a rich history dating back to the early 20th century, with Elie Metchnikoff suggesting that consumption of fermented dairy products contributed to the longevity of Bulgarian peasants. In the 1930s, Alfred Nissle developed a probiotic strain of *Escherichia coli* for gastrointestinal disorders. Today, probiotics are available in various forms and used for different conditions. Ongoing research has focused on their mechanisms of action and the development of NGPs (Figure 3a and b) for individual needs.^23^

**Figure 4:**
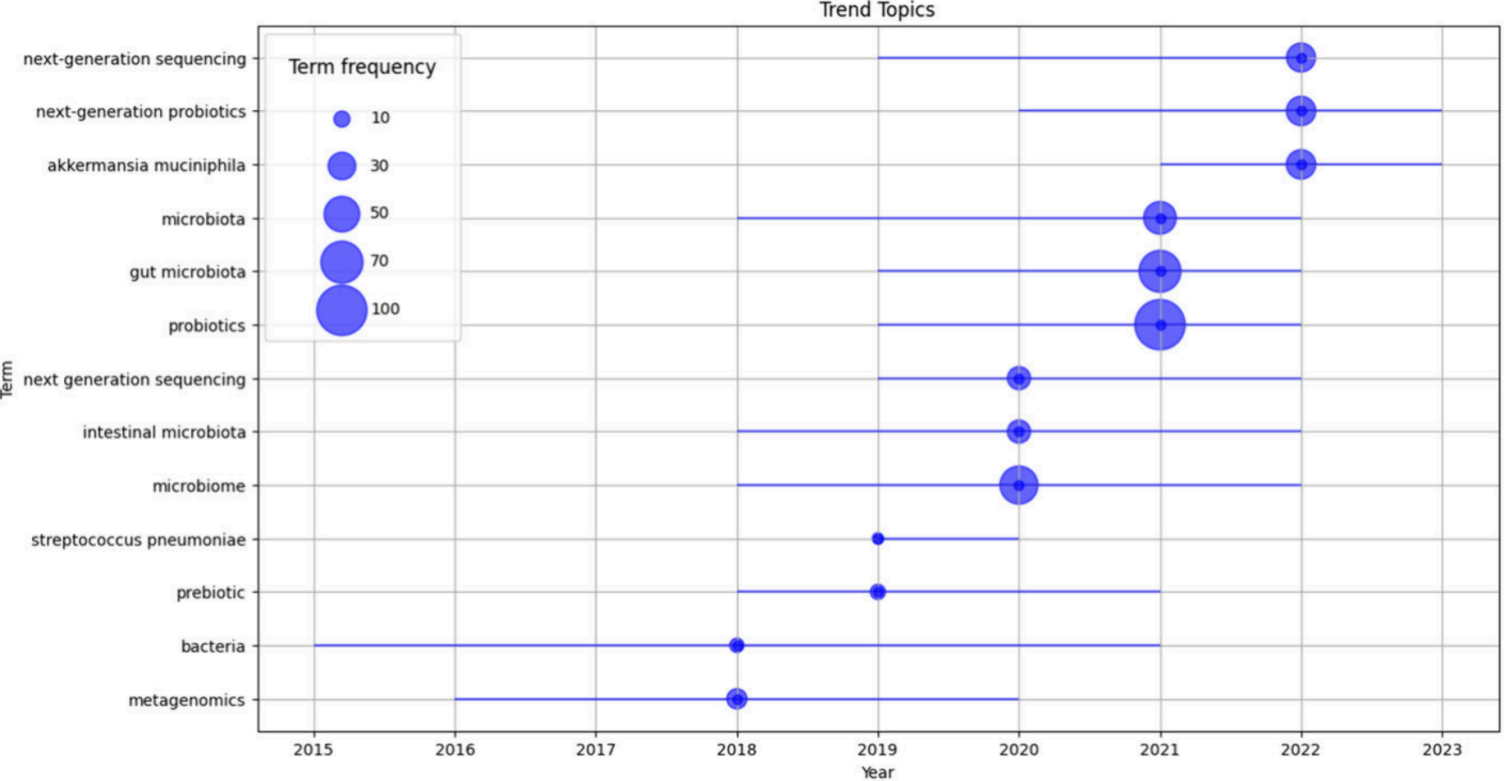
Trend analysis of key topics in probiotic research (2015–2023). This trend analysis visualizes the evolution of research topics in the field of probiotics from 2015 to 2023. The size of the bubbles indicates the frequency of each term in research publications, showing how interest in various topics has grown or shifted over time. This can help identify emerging trends and areas of increasing research focus within the probiotic research community.

Various types of probiotics, such as *Lactobacillus*, *Bifidobacterium*, *Streptococcus*, and *Saccharomyces*, are available in supplements, fermented foods, and dairy products (Table 2).^24,25^ The efficacy of probiotics varies depending on the specific strain, dosage, and delivery method, and some studies showed limited or no beneficial effects of probiotics against certain conditions.^26^ Limitations of current probiotics include their transient effects on the gut microbiome, lack of standardization, and potential safety concerns in certain populations (Table 3). Future research is needed to develop NGPs that can provide targeted therapeutic effects for specific conditions (Figure 4).^27^

**Table 2:** Probiotics and Their Applications in Various Diseases

probiotic species	application in disease	details and mechanisms	ref
*Lactobacillus rhamnosus*	inflammatory bowel disease (IBD)	enhances gut barrier function, reduces inflammation by modulating cytokine levels	(132)
*Bifidobacterium longum*	IBD, irritable bowel syndrome (IBS)	improves gut flora balance, reduces symptoms of diarrhea-predominant IBS, decreases zonulin levels	(133)
*Escherichia coli* Nissle 1917	IBD	genetically modified to produce anti-inflammatory cytokines, enhances epithelial healing	(132)
*Faecalibacterium prausnitzii*	IBD, metabolic disorders	produces butyrate which has anti-inflammatory properties and supports gut health	(134)
*Akkermansia muciniphila*	obesity, diabetes	improves insulin sensitivity, reduces inflammation, enhances gut barrier function	(135)
*Lactobacillus gasseri*	diabetes	produces glucagon-like peptide (GLP)-1, influences insulin production, controls hyperglycemia	(132)
*Lactococcus lactis*	cancer, diabetes	modified to produce interleukin (IL)-10 and proinsulin, reduces pancreatic cell damage, enhances immune responses	(135)
*Saccharomyces boulardii*	*Clostridium difficile* infection	produces proteins that neutralize *C*. *difficile* toxins, reduces inflammation and tissue damage	(136)
*Bacteroides fragilis*	cancer, IBD	modulates immune responses, produces polysaccharide A which influences T-cell regulation	(137)
*Roseburia* spp.	metabolic disorders, IBD	produces butyrate, improves gut barrier function, reduces inflammation	(138)
*Eubacterium hallii*	obesity, diabetes	produces propionate and butyrate, improves insulin sensitivity	(139)

**Table 3:** Different Types of Probiotics and Their Limitations

type of probiotic	common strains	efficacy	limitations	ref
*Lactobacillus*	*L. acidophilus*, *L. rhamnosus*^a^, *L. casei*^a^	may improve digestive health, immune function, and reduce risk of antibiotic-associated diarrhea	may not survive stomach acid, limited colonization, variability in efficacy	(140)
*Bifidobacterium*	*B. bifidum*, *B. lactis*, *B. longum*^a^	may improve gut health and immune function, and reduce inflammation	limited colonization, variability in efficacy	(141, 142)
*Streptococcus*	*S. thermophilus*	may improve lactose digestion and gut health	limited data on efficacy, potential for antibiotic resistance	(143)
*Saccharomyces*	*S. boulardii*	may improve digestive health and reduce risk of antibiotic-associated diarrhea and *Clostridium difficile* infection	limited colonization, variability in efficacy	(144)
*Escherichia*	*E. coli* Nissle 1917^a^	may improve digestive health and reduce risk of diarrhea	limited data on efficacy, potential for pathogenicity	(145)
*Bacillus*	*B. coagulans*	may improve digestive health and reduce risk of antibiotic-associated diarrhea	limited data on efficacy, potential for antibiotic resistance	(146)
*Enterococcus*	*E. faecium*, *E. faecalis*	may improve gut health and reduce inflammation	potential for pathogenicity, limited data on efficacy	(147, 148)
*Propionibacterium*	*P. freudenreichii*	may improve gut health and reduce inflammation	limited data on efficacy, potential for antibiotic resistance	(149)

a Probiotics with GIT survival challenges.

Research on the effects of probiotics on chronic diseases has shown promising results, particularly in the areas of gut health and immune function. Several clinical studies demonstrated the potential of probiotics to improve the gut barrier function, reduce inflammation, and modulate the gut microbiome.^28^ For example, a systematic review and meta-analysis of 15 randomized controlled trials found that probiotic supplementation significantly reduced markers of inflammation in individuals with metabolic disorders such as type 2 diabetes and obesity.^29^ Probiotics have also shown potential in preventing and treating CVDs. A meta-analysis of 13 randomized controlled trials found that probiotic supplementation significantly reduced blood pressure (BP) in hypertensive individuals. Similarly, another meta-analysis of 26 randomized controlled trials found that probiotics significantly reduced serum cholesterol levels, particularly low-density lipoprotein (LDL) cholesterol, in individuals with hypercholesterolemia.^30,31^ Inflammatory bowel disease (IBD), which includes Crohn’s disease and ulcerative colitis, is another area of interest for probiotic research. Several studies have shown that probiotics may help reduce inflammation and improve symptoms in individuals with IBD. For example, a randomized controlled trial of 116 patients with ulcerative colitis (UC) found that probiotic supplementation significantly reduced disease activity and improved quality of life compared to a placebo.^32^ Despite these promising findings, the use of probiotics for preventing and treating chronic diseases is not without limitations. The efficacy of probiotics can vary depending on the specific strain, dosage, and delivery method, and their safety in certain populations, such as immunocompromised individuals, is not well-established.^33^ Also, the mechanisms by which probiotics exert their effects on chronic diseases are not fully appreciated, and further research ought to elucidate these mechanisms.^34^

## 3 Exploring Next-Generation Probiotics and Chronic Diseases

NGPs refer to new and improved strains of beneficial bacteria that have been developed through advanced technologies, such as genomics and metagenomics. These probiotics are designed to offer superior health benefits compared to traditional probiotics by targeting specific health conditions and exhibiting unique characteristics, such as increased potency, stability, and survivability in the gut.^35^ They may also be engineered to produce specific compounds or molecules that have therapeutic properties, further enhancing their potential health benefits.^36,37^ In general, NGPs represent a promising avenue for improving human health and treating various diseases.^38^

Chronic diseases can be largely regarded as health conditions that last 1 year or more and require constant medical attention, limit regular activities, or both.^39^ NGPs are being developed to address limitations of current probiotics and provide more-effective treatment for chronic diseases. These new probiotics are designed to be more targeted and provide more-specific therapeutic effects for certain conditions. One approach to developing NGPs is to engineer specific strains to express therapeutic molecules or proteins. For example, *Lactobacillus* bacteria can be engineered to produce anti-inflammatory compounds such as interleukin (IL)-10 or transforming growth factor (TGF)-β, which can help reduce inflammation in the gut and alleviate symptoms of IBD.^40,41^ Similarly, *Bifidobacterium* strains can be engineered to produce peptides that have antimicrobial properties, which can be used to treat infections. The second approach is to use probiotics as delivery vehicles for targeted therapies. For example, *Lactobacillus* bacteria can be used to deliver therapeutic genes to the gut mucosa, which can be used to treat conditions such as colon cancer or UC.^42−44^ Also, engineered probiotics can be used to deliver specific drugs or vaccines to the gut, which can improve their efficacy and reduce side effects. NGPs can also be tailored to individual needs based on an individual’s gut microbiome. Personalized probiotics can be designed to restore or modulate specific bacterial populations in the gut that are associated with diseases.^45^ For example, probiotics can be designed to promote the growth of certain bacteria that are associated with improved metabolic health, which can be used to treat conditions such as obesity or type 2 diabetes. NGPs hold great promise for treating chronic diseases.^46^ However, further research is needed to determine their safety and efficacy, as well as to develop more efficient and cost-effective methods for their production and delivery (Table 4).

**Table 4:** Main Characterizations, Functions, Mechanisms, and Potential Weaknesses of Next-Generation Probiotics

next-generation probiotics	main characterizations	functions and mechanisms	potential weaknesses	ref
*Akkermansia muciniphila*	anaerobic gram-negative bacterium, abundant in the human gut	enhances gut barrier function, regulates inflammation, promotes metabolic health	limited availability, oxygen sensitive	(150)
*Faecalibacterium prausnitzii*	anaerobic gram-positive bacterium, commensal in the human gut	has anti-inflammatory effects, improves gut barrier function, produces short-chain fatty acids	difficult to culture, highly sensitive to oxygen	(151)
*Bifidobacterium lactis* HN019	anaerobic gram-positive bacterium, naturally found in the human gut	reduces inflammation, improves immune function, promotes digestive health	effectiveness may vary among individuals	(152)
*Lactobacillus acidophilus* NCFM	facultative anaerobic gram-positive bacterium, commonly used in probiotic products	enhances immune function, improves digestive health, may have anti-inflammatory effects	varying efficacy in different individuals	(153)
*Lactobacillus plantarum*	facultative anaerobic gram-positive bacterium, found in many fermented foods	improves gut barrier function, regulates inflammation, enhances immune function	inconsistent results across different populations	(154)
*Streptococcus thermophilus*	facultative anaerobic gram-positive bacterium, commonly used in dairy products	enhances lactose digestion, may have anti-inflammatory effects, improves immune function	potential variability in effectiveness	(155)
*Bacteroides uniformis*	anaerobic gram-negative bacterium, found in the human gut	enhances gut barrier function, improves immune function, may have anti-inflammatory effects	difficult to maintain, oxygen sensitive	(156)
*Parabacteroides distasonis*	anaerobic gram-negative bacterium, found in the human gut	improves gut barrier function, enhances immune function, may have anti-inflammatory effects	challenging to culture, oxygen sensitive	(157)

### 3.1 Approaches and Potential in Developing Next-Generation Probiotics

NGPs represent a promising frontier in medical research, aiming to create more effective and targeted probiotic therapies. These probiotics are designed to provide specific health benefits by modulating the gut microbiome, enhancing the efficacy of traditional probiotics, and offering new therapeutic options for chronic diseases. Unlike general probiotics, which provide conventional benefits by supporting gut health, NGPs are designated to address specific health benefits by modulating the gut microbiome. Through targeted interaction with key microbial pathways, NGPs are tailored to enhance traditional probiotic efficacy, creating the way for innovative treatments for various chronic diseases, including autoimmune diseases, metabolic disorders and gastrointestinal conditions (Table 5). Several innovative approaches are being explored to develop these NGPs, including synthetic biology, CRISPR/Cas9 gene editing, and gut microbiome engineering (Table 6).^47^

**Table 5:** Engineered Probiotics and Live Biotherapeutics Targeting Diseases

probiotic/biotherapeutic	engineered characteristics	target diseases	mechanisms of action	ref
*Lactococcus lactis*	secretes interleukin (IL)-10	Crohn’s disease	anti-inflammatory cytokine production	(158)
*Escherichia coli* Nissle 1917	produces human insulin	diabetes	insulin production to regulate blood glucose levels	(159)
*Bacteroides ovatus*	secretes prodrug enzyme	colorectal cancer	conversion of the prodrug to an active drug at tumor sites	(160)
*Lactobacillus reuteri*	produces oxytocin	autism spectrum disorders	enhances social behavior and reduces anxiety	(161)
*Clostridium butyricum*	produces butyrate	metabolic disorders (e.g., obesity, diabetes)	modulates the gut microbiota and improves metabolism	(162)
*E. coli* Nissle 1917	secretes angiogenin	ischemic stroke	promotes angiogenesis and neuroprotection	(163)
*Bifidobacterium longum*	expresses l-arginine	ulcerative colitis	enhances mucosal healing and immune modulation	(164)
*Lactobacillus plantarum* EJ2014	produces GABA	anxiety and depression	modulates neurotransmitter levels	(165)
*E. coli* Nissle 1917	produces antimicrobial peptides	infections (e.g., urinary tract infections)	directly kills pathogenic bacteria	(166)
*Bacteroides fragilis*	expresses polysaccharide A (PSA)	central nervous system demyelinating disease	modulates immune responses and reduces inflammation	(167)
*Lactobacillus casei*	secretes bile salt hydrolase	hypercholesterolemia	reduces cholesterol levels by deconjugating bile acids	(168)
*E. coli* Nissle 1917	produces 5-aminosalicylic acid	ulcerative colitis	has anti-inflammatory effects	(169)
*Lactobacillus johnsonii*	produces exopolysaccharides	atopic dermatitis	strengthens the gut barrier and modulates immune response	(170)
*Lactococcus lactis*	secretes tumor necrosis factor receptor (TNF-R)	chronic colitis	neutralizes TNF-α to reduce inflammation	(171)
*E. coli* Nissle 1917	expresses glucagon-like peptide (GLP)-1	type 2 diabetes	enhances insulin secretion and reduces blood glucose levels	(172)
*Bifidobacterium breve*	produces folate	folate deficiency	increases folate levels in the body	(173)
*Lactobacillus gasseri*	produces α-galactosidase	irritable bowel syndrome	reduces symptoms by breaking down complex carbohydrates	(174)
*E. coli* Nissle 1917	produces anti-inflammatory cytokines	Crohn’s disease	reduces intestinal inflammation	(175)
*Lactococcus lactis*	secretes leptin	obesity	regulates appetite and energy balance	(176)
*Lactobacillus paracasei*	produces S-layer protein A	*Helicobacter pylori* infection	inhibits *H. pylori* adhesion to gastric mucosa	(177)
*Streptococcus salivarius*	produces bacteriocin BLIS K12	oral infections (e.g., strep throat, dental caries)	inhibits growth of pathogenic oral bacteria	(178)
*E. coli* Nissle 1917	produces nitric oxide	hypertension	vasodilation and blood pressure reduction	(179)
Lactobacillus rhamnosus*	produces lactase	lactose intolerance	enhances lactose digestion	(180)
*E. coli* Nissle 1917	produces arginine deiminase	cancer	starves tumor cells of arginine	(181)
*Lactobacillus jensenii*	expresses cyanovirin-N	human immunodeficiency virus (HIV) prevention	inhibits HIV entry into host cells	(182)
*Lactobacillus acidophilus*	produces reuterin	gastrointestinal infections	promotes antimicrobial activity against pathogens	(183, 184)
*Bacillus subtilis*	produces nattokinase	cardiovascular diseases (e.g., thrombosis)	breaks down fibrin and prevents blood clots	(185)
*Lactococcus lactis*	secretes superoxide dismutase (SOD)	oxidative stress-related diseases	reduces oxidative stress and damage	(186)
*Lactobacillus casei* Shirota	produces short-chain fatty acids	colitis	has anti-inflammatory properties and protects the gut barrier	(187)

**Table 6:** Comprehensive Table of Genetic Engineering Tools Tailored for Building Next-Generation Probiotics (NGPs)

tools	description	application	example organism	ref
CRISPR-Cas9	a precise genome-editing tool that can introduce targeted double-strand breaks in DNA	gene knockout, gene insertion, gene regulation, and genome-wide screening	*Lactobacillus reuteri* for enhancing probiotic traits.	(188)
TALENs	transcription activator-like effector nucleases (TALENs) that can be designed to target specific DNA sequences	gene editing, gene disruption, and gene integration	*Bifidobacterium longum* for modifying metabolic pathways.	(189)
zinc finger nucleases (ZFNs)	customizable DNA-binding proteins that create double-strand breaks at specific genomic locations	gene editing, gene disruption, and gene integration	*Escherichia coli* for creating probiotic strains with therapeutic capabilities.	(190)
recombinase systems (e.g., Cre-Lox)	enzyme-based systems that mediate site-specific recombination between DNA sequences	conditional gene knockout, gene activation, and genome rearrangement	*Bacillus subtilis* for controlled gene expression in probiotics.	(191)
integrative and conjugative elements (ICEs)	mobile genetic elements that can be integrated into a host genome and facilitate horizontal gene transfer	introducing and spreading beneficial traits among microbial populations	*Lactococcus lactis* for introducing beneficial traits.	(192)
plasmids	circular DNA molecules used as vectors for gene cloning and expression	cloning, gene expression, and protein production	*Lactobacillus acidophilus* for expressing therapeutic proteins.	(193)
lambda red recombination	a phage-derived system for homologous recombination in bacteria	genome editing and gene knock-in in prokaryotes	*Escherichia coli* for precise genetic modifications in probiotic research.	(194)
synthetic biology circuits	engineered gene networks that can perform logical functions and control gene expression	biosensing, metabolic engineering, and programmable gene expression	*Escherichia coli* engineered to detect and respond to gut inflammation.	(195)
genome shuffling	a method to accelerate the evolution of microbial strains through recombination of whole genomes	improving complex traits such as stress resistance and metabolic pathways	*Saccharomyces cerevisiae* for enhanced probiotic properties.	(196)
RNA interference (RNAi)	a technique to silence gene expression by degrading specific mrna molecules	gene knockdown and functional genomics studies	*Saccharomyces cerevisiae* for studying gene function in probiotic strains.	(197)
transposon mutagenesis	insertion of transposons into the genome to disrupt gene function	functional genomics, gene discovery, and strain improvement	*Lactobacillus plantarum* for identifying genes involved in probiotic functions.	(198)
phage display	a technique to study protein interactions by expressing proteins on the surface of bacteriophages	identifying protein–protein interactions, antibody engineering, and peptide screening	*Escherichia coli* phages for developing therapeutic peptides.	(199)
metabolic engineering	modification of metabolic pathways to improve production of desired compounds	production of biofuels, pharmaceuticals, and other valuable chemicals	*Lactobacillus casei* for enhanced production of health-promoting metabolites.	(200)
adaptive laboratory evolution (ALE)	a method to evolve microbial populations under controlled conditions to acquire desirable traits	improving strain robustness, metabolic efficiency, and environmental tolerance	*Escherichia coli* for developing stress-resistant probiotic strains.	(201)
single-cell genomics	analyzing the genome of individual cells to understand microbial diversity and function	studying microbial populations, identifying rare genotypes, and understanding gene expression variability	various gut microbiota species for probiotic research.	(202)

### 3.2 Examining the Role of Synthetic Biology

The gut microbiota has an integral role in host metabolism through many intricate interactions with systems, including the digestive, immune and nervous systems. Synthetic biology involves designing and constructing new biological systems with desired properties, including bacteria that can be used as probiotics. This approach allows scientists to create bacterial strains that produce specific metabolites or proteins or that are more resistant to harsh environmental conditions. For instance, bacteria can be engineered to produce SCFAs, which have anti-inflammatory properties and can enhance gut health.^48^ Advances in synthetic biology have opened new ways for the alteration of probiotics, transforming them into specific, smart therapeutics capable of detecting and responding to host signals and delivering treatments in a controlled manner. Selection of an appropriate chassis for engineering probiotics is important as the chosen microbial chassis must be modifiable, compatible with genetic tools, and capable of effective colonization. Most commonly available chassis include *E. coli, Lactobacillus, Bifidobacterium* and new candidates such as *Bacteroides*, *Clostridium butyricum*, and *Saccharomyces boulardii*. Each has their distinct advantages and challenges. Also, successful colonization is crucial for the functionality of engineered probiotics, certain methods like surface modifications involving encapsulation of probiotics with protective coatings such as polysaccharides or silk fibroin to enhance the adhesion and survival in gastrointestinal tract. Genetic enhancements and competitive niche strategies have been used.^49^

An example of synthetic biology in action is the engineering of *Lactobacillus paracasei* to produce conjugated linoleic acid (CLA), a compound known to promote weight loss in animal models. Researchers modified the genetic makeup of *L. paracasei* to increase its production of CLA, resulting in a probiotic that when administered to obese mice, significantly reduced body weight and fat mass, while improving glucose metabolism and insulin sensitivity.^50,51^

Another example is the engineering of *E. coli* to produce the anorexigenic hormone, peptide YY (PYY), which suppresses appetite. This engineered *E. coli* strain, when given to obese rats, led to reduced food intake, body weight, and fat mass, demonstrating the potential of synthetic biology to create probiotics with specific therapeutic functions.^52,53^

### 3.3 Utilizing CRISPR/Cas9 Gene Editing

The discovery of Clustered Regularly interspaced Short Palindromic Repeats (CRISPR) and its applications as a powerful gene editing tool has revolutionized molecular biology. It allows for precise genetic modifications, which can be used to enhance the functionality of probiotics. Using CRISPR/Cas9, scientists can insert, delete, or modify specific genes within a probiotic strain to enhance its ability to survive in the gut or produce beneficial compounds with enhanced therapeutic potential.^54^

The CRISPR-Cas systems can be classified into class 1 (multiprotein complexes) and Class 2 (single-protein effectors) systems, where Class 2 systems being widely used in genome editing because of their simplicity. The type 2 CRISPR-Cas9 system, from *Streptococcus pyogenes* (SpyCas9), is one of the most used for genetic engineering. Other variants such as Cas12a (Cpf1) have extended the CRISPR toolbox, offering alternative protospacer adjacent motif (PAM) requirements and cleavage patterns. This technology has been widely implemented in various bacteria including *E. coli* and *Lactobacillus* species, supporting precise genetic alterations such as insertions, deletions and point mutations.^55^

For example, researchers have used CRISPR/Cas9 to delete genes in *Lactobacillus* species that make them sensitive to stomach acid, thereby increasing their survival rate through the digestive tract.^56^ Additionally, CRISPR/Cas9 has been used to insert genes into *Bifidobacterium* strains that allow them to produce anti-inflammatory molecules, improving their therapeutic potential for conditions like IBD.^57^

In a study involving *Akkermansia muciniphila*, a bacterium known for its beneficial effects on metabolic health, CRISPR/Cas9 was used to enhance its ability to produce mucin-degrading enzymes. These enzymes improve the bacterium’s ability to strengthen the gut barrier and reduce inflammation, making it more effective in treating metabolic disorders like diabetes and obesity.^58^

CRISPR-Cas9 technology possesses powerful applications for engineering probiotics to improve their therapeutic potential. The use of CRISPR-Cas9 in probiotics can be applied from several angles, to enhance probiotic properties by improved stress tolerance and stability by using CRISPR-Cas9 to overexpress genes related to stress response, such as molecular chaperons and osmoprotectant systems increasing their survival and effectiveness in the host. Also, the introduction or modification of metabolic pathways to expand their utilization of dietary compounds can be achieved by CRISPR-Cas9 system. Another angle to this approach includes- mucosal vaccine delivery by displaying viral epitopes on their cell surface, enhancing anti-inflammatory and immunomodulatory properties of probiotics, elimination of antibiotic resistance genes, development of disease specific engineered probiotic strains.^59^

Despite the promising applications, the use of CRISPR-Cas9 in next generation probiotic engineering faces challenges. The efficiency of homology-directed repair (HDR) in many probiotic strains is often low, which can limit editing capabilities of certain probiotics. Nevertheless, strategies such as applying Cas9 nickase variants and base editors can circumvent these limitations, enabling the possibility of targeted modifications without double stranded DNA breaks.^60^

### 3.4 Strategies for Gut Microbiome Engineering

The role of the gut microbiome in human health and disease is widely recognized, as discussed in previous sections. Engineering single microbial strains often proves inadequate suggesting that creating a supportive and collaborative microbial network, termed a “keystone consortium” could be more effective. Here, we have summarized various gut microbiome engineering strategies. Microbiome engineering is predominantly applied to the human microbiome due to its potential for disease treatment also the launch of Human Microbiome Project (HMP) has greatly enhanced the characterization of human microbiota across the gut and other regions. Enabling identification of differences between healthy and diseased microbiota and helping in devising therapeutic strategies to restore gut microbiome balance.^61^

Gut microbiome engineering involves modifying the composition and function of the gut microbiome to promote health and prevent disease. This approach can include introducing specific probiotic strains to the gut or altering the existing microbial community to achieve desired health outcomes.^62^

One method of gut microbiome engineering is fecal microbiota transplantation (FMT), where the fecal matter from a healthy donor is transplanted into the gut of a patient. This was shown to be effective in treating *Clostridium difficile* infections (CDI) and is being explored for other conditions, such as IBD and metabolic syndrome. Whereas traditional antibiotic treatment for CDI can lead to antibiotic resistance and disrupt gut microbiota, FMT helps restore beneficial microbes, achieving high cure rates and low recurrence. While FMT is not a probiotic therapy per se, it highlights the potential of microbiome engineering to significantly alter gut health.^63^ Other microbiome engineering strategies investigated in animal models include the use of antimicrobial peptides (AMPs) like thuricin CD and pyocin S5 for targeted pathogen elimination with minimal disturbance to gut microbiota. Microbiome engineering outside the GI tract remains less explored. However, there are promising studies, such as using probiotics like *Lactobacillus* to support beneficial skin bacteria and inhibit pathogenic ones, potentially treating conditions like atopic dermatitis. Engineering the oral microbiome with AMPs like C16G2 to target *Streptococcus mutans*, a dental decay-causing bacterium, has been effective. Research into lung microbiomes is still developing, with studies focusing on differentiating between healthy and diseased states to identify engineering targets.^64^

Probiotics can also be used to introduce beneficial bacterial strains that are underrepresented in the gut microbiome of individuals with certain diseases. For example, *Faecalibacterium prausnitzii*, an anaerobic bacterium known for its anti-inflammatory effects,^65^ can be administered to patients with IBD to help reduce inflammation and improve gut barrier function. Similarly, *Bacteroides uniformis*, which enhances gut barrier function and immune responses, can be used to support patients with gut-related health issues.^66^ Engineering the gut microbiome holds significant potential for treating various diseases that offers opportunities to develop innovative therapies.

## 4 Assessing the Potential of Next-Generation Probiotics for Managing Chronic Diseases

NGPs have the potential to address a wide range of chronic diseases, characterized by long-term, persistent health conditions that require ongoing management and treatment, such as CVDs, diabetes, and IBD (Table 7). Studies have shown that NGPs can help alleviate symptoms and improve outcomes for people with chronic diseases.^67^ For example, a specific strain of NGPs was able to improve insulin sensitivity in rats with type 2 diabetes.^68^ Another study found that an NGP containing multiple strains was able to reduce symptoms in people with IBD.^69^ NGPs may also have a role in preventing chronic diseases. For instance, research showed that certain strains of probiotics can help reduce cholesterol levels and improve cardiovascular health, potentially preventing the development of CVDs.^70^

**Table 7:** Next-Generation Probiotics and Their Potential Benefits for Chronic Diseases

chronic disease	next-generation probiotic	potential benefits
inflammatory bowel disease	*Akkermansia muciniphila*	reduces inflammation, strengthens the gut barrier
	*Faecalibacterium prausnitzii*	lowers inflammation, enhances gut barrier function
	*Bifidobacterium longum*	minimizes inflammation, fortifies the gut barrier
	*Escherichia coli* Nissle 1917	diminishes inflammation, improves the intestinal barrier
type 2 diabetes	*Bifidobacterium lactis*	enhances glucose metabolism, reduces inflammation
	*Lactobacillus acidophilus*	boosts glucose metabolism, decreases inflammation
	*Akkermansia muciniphila*	optimizes glucose metabolism, mitigates inflammation
	*Faecalibacterium prausnitzii*	improves glucose metabolism, alleviates inflammation
	*Streptococcus thermophilus*	regulates glucose metabolism, lowers inflammation
cardiovascular diseases	*Lactobacillus reuteri*	lowers cholesterol levels, reduces blood pressure
	*Bifidobacterium bifidum*	decreases inflammation, lowers blood pressure
	*Lactobacillus plantarum*	reduces inflammation, improves endothelial function
	*Lactobacillus acidophilus*	decreases inflammation, lowers cholesterol levels
	*Lactobacillus fermentum*	reduces inflammation, decreases cholesterol levels
obesity	*Lactobacillus paracasei*	promotes weight loss, improves glucose metabolism
	*E. coli* (engineered)	suppresses appetite, reduces body fat
	*Akkermansia muciniphila*	enhances metabolism, reduces body weight
	*Bifidobacterium breve*	decreases body fat, improves insulin sensitivity
	*Lactobacillus gasseri*	reduces abdominal fat, enhances lipid metabolism

NGPs offer numerous benefits in preventing and supporting treatments of chronic diseases. They can improve gut health by restoring the balance of beneficial bacteria, which helps prevent and treat GI disorders such as IBD, IBS, and constipation.^71^ They can enhance immune function, as the gut microbiome plays a crucial role in modulating the immune system, thereby reducing the risk of autoimmune diseases and infections.^72^ In addition, they can reduce chronic inflammation, which is associated with a wide range of chronic diseases, including CVDs, type 2 diabetes, and cancer.^73^ Moreover, there is growing evidence that the gut microbiome influences mental health, and NGPs may improve mental health outcomes by modulating the gut-brain axis.^74^ Personalized medicine is another promising area, where NGPs can be tailored to an individual’s specific gut microbiome composition and health needs, allowing for personalized treatment of chronic diseases.^75^

Studies have shown the potential of NGPs for treating IBD. One study demonstrated that an engineered probiotic secreting anti-inflammatory cytokines reduced inflammation in mouse models of colitis.^76^ Another study showed that a multispecies probiotic reduced disease activity in patients with active mild to moderate UC.^77^ For type 2 diabetes, NGPs were investigated as a potential treatment by improving glucose regulation and insulin sensitivity. One study showed that a probiotic combination improved insulin sensitivity and reduced inflammation in overweight and obese individuals with type 2 diabetes.^78^ For CVDs, a study demonstrated that a probiotic combination reduced LDL cholesterol and triglycerides in patients with hypercholesterolemia.^79^ Another study showed that a probiotic intervention reduced arterial stiffness in patients with metabolic syndrome.^80^

### 4.1 Exploring Next-Generation Probiotics for the Prevention of Chronic Diseases

**Figure 5:**
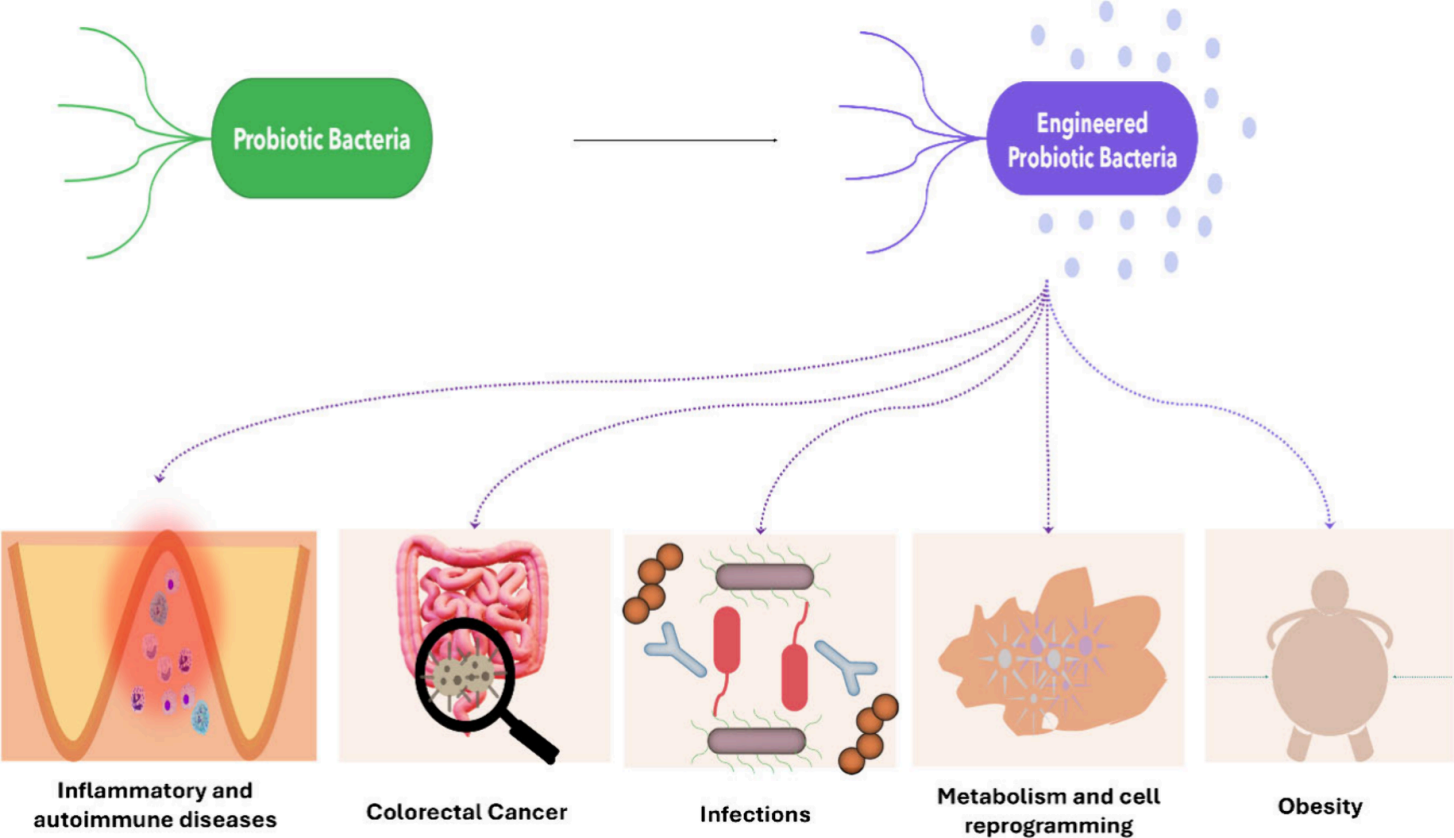
Potential therapeutic applications of engineered probiotic bacteria. This illustration explains the progression from traditional probiotic bacteria to engineered probiotics and their potential applications in treating a variety of health conditions. The use of engineered probiotics represents a significant advancement in the field, offering targeted therapeutic benefits beyond the general health advantages of traditional probiotics.

Chronic diseases such as diabetes, CVDs, and cancer are major health concerns globally. Although these diseases have multifactorial causes, emerging evidence suggests that the gut microbiome plays a critical role in their pathogenesis.^81^ Consequently, there is growing interest in potentially using NGPs as preventative measures for chronic diseases.^82^ NGPs are a new class of probiotics designed to target specific microbiotic strains or metabolic pathways to achieve therapeutic outcomes (Figure 5). These probiotics can be engineered using synthetic biology, gene editing, or other advanced technologies to enhance their efficacy and safety as discussed before. Several studies investigated the use of NGPs for preventing chronic diseases.^83^ For example, in a randomized, double-blind, placebo-controlled trial, an NGP containing *Lactobacillus reuteri* NCIMB 30242 supported treatment by significantly reducing LDL-cholesterol levels in patients with hypercholesterolemia.^84^ Another study indicated that an NGP containing *Lactobacillus crispatus* reduced the incidence of urinary tract infections in women with a history of recurrent infections.^85^ NGPs were also studied for their potential to prevent diabetes. In a preclinical study, an NGP containing a modified version of *Akkermansia muciniphila*, a gut bacterium associated with improved glucose metabolism, supported the prevention of diabetes onset in mice fed a high-fat diet.^86,87^ Another study suggested that an NGP containing *Bifidobacterium lactis* and *Lactobacillus acidophilus* improved insulin sensitivity and glucose metabolism in patients with type 2 diabetes.^88^ NGPs may also have a role in preventing cancer. In a mouse model of colorectal cancer,^89^ an NGP containing a recombinant strain of *Lactococcus lactis* expressing the IL-17A cytokine supported a reduction in tumor growth.^90^ Another study showed that an NGP containing *Lactobacillus rhamnosus* GG and *Bifidobacterium lactis* Bb12 reduced the incidence and severity of oral mucositis in patients undergoing chemotherapy for head and neck cancer.^91,92^ Despite these promising results, the use of NGPs for preventing chronic diseases is still in its infancy. More research is needed to establish their safety and efficacy, as well as to identify optimal strains and dosages for specific disease indications. In addition, regulatory frameworks for the development and commercialization of NGPs are still evolving.^93^ NGPs hold great promise as preventative measures for chronic diseases. However, further research is needed to fully understand their potential and limitations, as well as to establish a regulatory framework for their development and use.^94^ With continued advances in microbiome research and biotechnology, NGPs are likely to play an increasingly important role in preventing and treating chronic diseases in the future.

### 4.2 Investigating Next-Generation Probiotics for Diabetes

NGPs are being developed as potential treatments for various chronic diseases, including diabetes. Diabetes is a chronic metabolic disorder that affects millions of people worldwide. Despite significant progress in the development of various therapeutic strategies, diabetes management remains a major challenge.^95,96^ Probiotics have emerged as a promising therapeutic option for preventing and managing diabetes. Studies have shown that gut microbiota dysbiosis, characterized by an imbalance in the composition and diversity of the gut microbiota, plays a significant role in the pathogenesis of diabetes.^97^ The gut microbiota was shown to regulate host metabolism, insulin resistance, and inflammation, all of which are involved in the development and progression of diabetes. Thus, interventions that target the gut microbiota may represent a promising therapeutic approach for diabetes.^98^ NGPs were designed to modulate the gut microbiota to achieve specific therapeutic outcomes. They were engineered to target specific bacterial strains, produce specific metabolites, or express specific proteins that can modulate host metabolism and immune function.^99^ NGPs offer several advantages over traditional probiotics, including improved specificity, efficacy, and safety. Many studies have investigated the potential of NGPs for preventing and treating diabetes.^100^ For example, *Bifidobacterium adolescentis* SPM0212 supported treatment by improving glucose tolerance and insulin sensitivity in a mouse model of type 2 diabetes. The probiotic was found to modulate the gut microbiota and reduce inflammation, which are key mechanisms underlying the development of insulin resistance and diabetes.^101^ Another study indicated that *Lactobacillus curvatus* HY7601 and *Lactobacillus plantarum* KY1032 improved glucose tolerance and insulin sensitivity in a high-fat diet-induced diabetic mouse model.^102^ The probiotic was found to modulate the gut microbiota and increase the production of SCFAs, which are known to improve glucose metabolism and insulin sensitivity. Additionally, a study investigating the effects of *Akkermansia muciniphila* on glucose metabolism and insulin sensitivity in overweight and obese human subjects found that the probiotic improved insulin sensitivity and reduced inflammation, suggesting that it may have potential as a therapeutic intervention for diabetes.^103^ Overall, those studies suggested that NGPs hold promise as potential treatments for diabetes. However, further research is needed to fully understand their mechanisms of action and to optimize their therapeutic potential. In addition, the safety and long-term effects of NGPs on human health require further investigation. NGPs represent an exciting area of research for preventing and treating chronic diseases such as diabetes. These probiotics offer several advantages over traditional probiotics and have shown promising results in preclinical and clinical studies. With further research, NGPs may become an important tool for managing diabetes and other metabolic disorders.

### 4.3 Supporting the Treatment of Obesity with Next-Generation Probiotics

Obesity is a chronic condition affecting millions of people worldwide and is associated with various health risks, including diabetes, CVDs and certain cancers. Despite numerous efforts to combat obesity, the prevalence of this condition continues to rise, underscoring the need for new and innovative approaches for its prevention and treatment. In recent years, there has been growing interest in the potential use of NGPs to prevent and treat obesity.^104^

Probiotics confer health benefits on the host. Traditionally, probiotics have been used to improve digestive health, boost the immune system, and treat certain infections. NGPs, on the other hand, are engineered to target specific metabolic pathways in the gut microbiome and may hold promise as a therapeutic approach for obesity. One potential mechanism by which NGPs could combat obesity is by altering the composition of the gut microbiota. Studies have shown that the gut microbiota of obese individuals differs from that of lean individuals, with a lower abundance of certain beneficial bacteria and an overgrowth of potentially harmful bacteria.^105,106^ By introducing specific strains of beneficial bacteria, NGPs could help restore a healthy balance of gut bacteria, thereby promoting weight loss and reducing the risk of obesity-related diseases. Another potential mechanism of action for NGPs in treating obesity is through the modulation of a host’s metabolism.^107^ Research showed that the gut microbiome plays a critical role in regulating host energy metabolism, and alterations in the gut microbiome were linked to the development of obesity and related metabolic disorders.^108^ NGPs designed to modulate specific metabolic pathways in the gut could therefore help regulate host energy metabolism and promote weight loss.^109^

Studies have investigated the potential of NGPs for treating obesity. In one study, researchers engineered a strain of *Lactobacillus paracasei* to produce a conjugated linoleic acid (CLA) isomer known to promote weight loss in animals.^110^ Administering this strain to obese mice resulted in significant reductions in body weight and fat mass, as well as improvements in glucose metabolism and insulin sensitivity. In another study, researchers developed an NGP using a strain of *E. coli* engineered to produce a potent anorexigenic hormone called peptide tyrosine tyrosine (PYY). When this strain was administered to obese rats, it resulted in reduced food intake, body weight, and fat mass, as well as improvements in glucose metabolism and insulin sensitivity.^111^ While those studies provided promising results, it is important to note that NGPs for treating obesity are still in the early stages of development, and much more research is needed before they can be used as a clinical treatment. Additionally, the safety and efficacy of these probiotics in humans need to be established through rigorous clinical trials.

NGPs hold promise as a therapeutic approach for preventing and treating obesity. By altering the composition of the gut microbiota and modulating a host’s metabolism, these probiotics could help promote weight loss and reduce the risk of obesity-related diseases. Further investigation is needed to establish the safety and efficacy of these probiotics, but the development of NGPs for treating obesity represents an exciting new area of research in the fight against this chronic condition.

### 4.4 Treating Hypertension with Next-Generation Probiotics

Hypertension, or high blood pressure (BP), is a major risk factor for CVDs and is a leading cause of morbidity and mortality worldwide. Despite the availability of various antihypertensive medications, many patients with hypertension do not achieve adequate BP control, highlighting the need for new therapeutic strategies.^112^ Recent research showed that the gut microbiota plays an important role in regulating BP through the production of bioactive compounds, such as SCFAs and nitric oxide. Therefore, NGPs targeting the gut microbiota represent a promising approach for treating hypertension.^113^

One such NGP is *Akkermansia muciniphila*, a Gram-negative bacterium that was shown to improve glucose metabolism and reduce inflammation in preclinical studies. In a randomized, double-blind, placebo-controlled trial, daily oral administration of *A. muciniphila* for 3 months significantly reduced systolic BP in overweight or obese adults with untreated hypertension.^103^ Another potential candidate for the development of NGPs for treating hypertension is *Lactobacillus plantarum*. In animal studies, oral administration of *L*. *plantarum* reduced BP, improved endothelial function, and decreased inflammation.^114^ In addition, engineered probiotics that produce angiotensin-converting enzyme (ACE) inhibitors, such as *Lactococcus lactis*, have shown potential for treating hypertension. ACE inhibitors are commonly used antihypertensive medications that block the production of angiotensin II, a potent vasoconstrictor.^115^

Regulatory approval for such therapies is likely to be challenging, as they would be considered a new class of biologic drugs. Nevertheless, engineered probiotics that produce ACE inhibitors represent an exciting area of research with the potential to revolutionize hypertension treatment. NGPs targeting the gut microbiota have shown promising results for treating hypertension. Further research is needed to elucidate the underlying mechanisms of action and optimize the efficacy and safety of these novel therapeutics.^116^

## 5 Challenges and Opportunities of Next-Generation Probiotics in Clinical Practice

**Figure 6:**
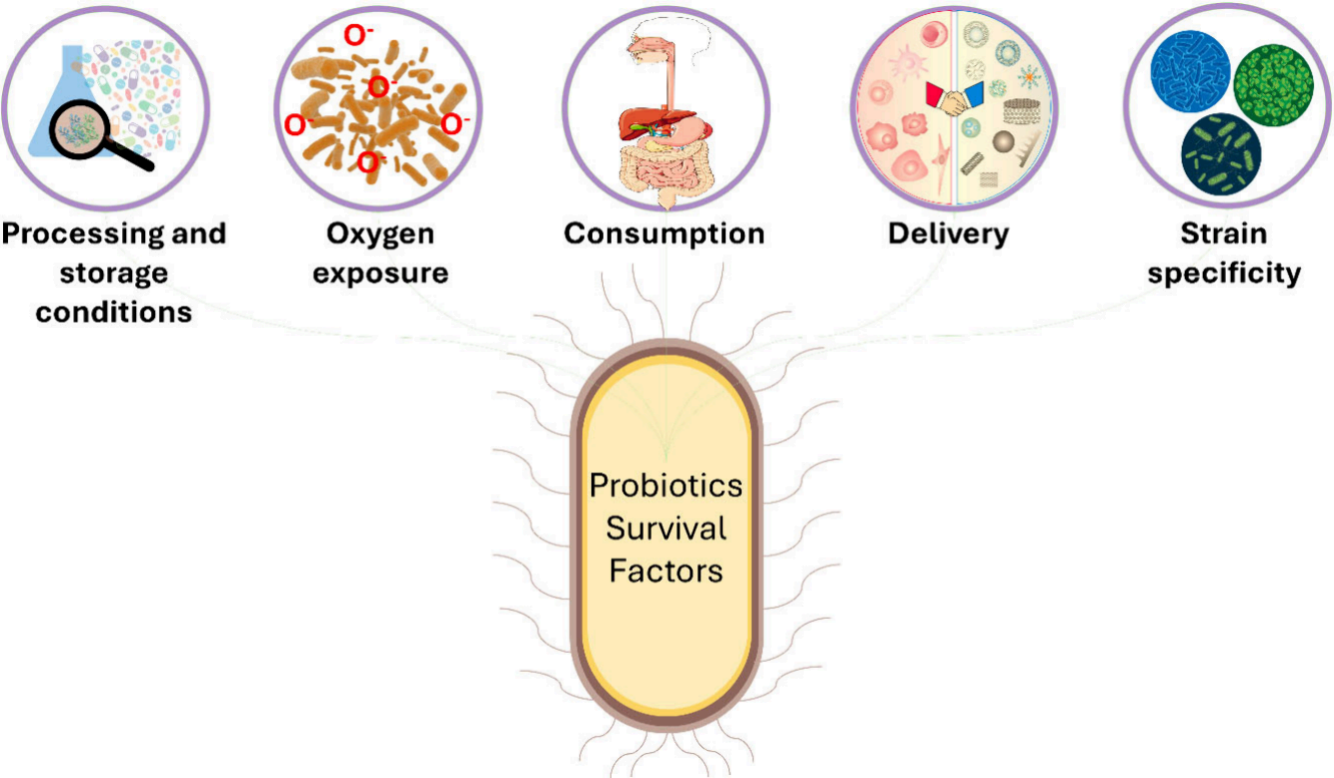
Key factors influencing probiotic survival. This diagram highlights the various factors that influence the survival and efficacy of probiotics from production to consumption. Understanding and optimizing these factors are essential to ensure that probiotics can provide their intended health benefits.

The use of NGPs in clinical practice presents both challenges and opportunities (Table 8). One challenge is the need for more-extensive research to demonstrate their efficacy and safety. While there have been promising results from preclinical studies and early phase clinical trials, larger and more rigorous studies are needed to establish the effectiveness of NGPs for treating specific chronic diseases. Another challenge is the complexity of the gut microbiome and the need to consider individual differences in microbial compositions and functions (Figure 6). This means that personalized approaches may be necessary to optimize the use of NGPs.^117^

**Table 8:** Challenges and Opportunities of Next-Generation Probiotics

challenges	opportunities
regulatory hurdles in bringing new probiotics to market	development of personalized probiotics based on individual microbiomes
difficulty in selecting an appropriate strain or combination of strains for a specific condition	greater understanding of the mechanisms of probiotics and the microbiome
limited understanding of the long-term effects of probiotic use	potential for probiotics to be used as low-cost, low-risk treatment options
lack of standardization in probiotic products and dosages	advances in technology, such as CRISPR/Cas9, for designing and engineering probiotics
risk of adverse effects in certain populations, such as immunocompromised individuals	potential to reduce antibiotic use and associated risks, such as antibiotic resistance

The innovative approaches in encapsulating probiotics using various hydrocolloids, which address some of the critical challenges in the clinical application of next-generation probiotics (NGPs). One of the primary challenges in utilizing NGPs is ensuring their stability and viability through the gastrointestinal tract, where they encounter harsh conditions like low pH and bile salts. The encapsulation techniques detailed in the table, such as using konjac glucomannan hydrogel or spray-dried succinate-grafted alginate, provide protective matrices that enhance the survivability of probiotics until they reach their target site.^118^ This encapsulation also allows for controlled and targeted release, which is crucial for the therapeutic efficacy of NGPs in treating specific diseases (Table 9).

**Table 9:** Hydrocolloid-Based Microencapsulation Methods for Probiotic Delivery

probiotic	hydrocolloid delivery method	ref
*Lactobacillus acidophilus*	microencapsulation with konjac glucomannan hydrogel	(203)
*Bifidobacterium bifidum*	microencapsulation in pectin-based gels	(204)
*Saccharomyces boulardii*	encapsulation in whey-protein isolate	(205)
*Lactobacillus rhamnosus* GG	encapsulation in whey protein isolate-shortening oil and gum arabic by complex coacervation	(206)
*Streptococcus thermophilus*	*Melastoma dodecandrum*-loaded chitosan-coated nanonutriosomes fruits extract (MDLE) for targeted delivery	(207)
*Lactobacillus casei*	alginate-based microgels for stability enhancement	(208)
*Bifidobacterium longum*	soy protein isolate-carrageenan conjugates microcapsules for controlled release	(209)
*Lactobacillus plantarum*	microencapsulation of soy hull polysaccharide concentration on stabilized high-internal-phase emulsions	(210)
*Bacillus coagulans*	starch- and carboxymethylcellulose-coated bacterial nanocellulose-pectin bionanocomposite for thermal stability	(211)
*Akkermansia muciniphila*	spray-dried succinate-grafted alginate doped with epigallocatechin-3-gallate based microcapsules for enhanced viability	(212)
*Faecalibacterium prausnitzii*	layer-by-layer encapsulation using riboflavin-conjugated sodium alginate and glycol chitosan encapsulation for anaerobic stability	(213)
*Lactococcus lactis*	poly(vinyl alcohol) films microcapsules for increased viability	(214)
*Pediococcus pentosaceus* Li05	encapsulation in microgels doped with inorganic nanoparticles	(215)
*Lactobacillus salivarius*	sugar beet pectin (SBP) as solid/oil/water emulsions.	(216)
*Lactobacillus helveticus*	microencapsulation in water-in-water Pickering emulsion composites	(217)
*Bifidobacterium animalis* F1–7	supramolecular hydrogel sodium alginate/tryptophan-Sulfobutylether-β-cyclodextrin (Alg/Trp-SBE-β-CD) for enhanced stability	(218)

On the other hand, there are several opportunities associated with NGPs. They have the potential to provide more targeted and specific interventions than traditional probiotics, which may increase their effectiveness. Additionally, the ability to engineer probiotics with specific functions and mechanisms could lead to new therapeutic approaches for chronic diseases. Furthermore, NGPs may have fewer side effects than traditional therapies, such as pharmaceuticals, which could improve patient adherence and quality of life (Table 10). Finally, the use of NGPs could lead to cost savings for healthcare systems by reducing the need for more-expensive treatments and hospitalizations. Overall, while there are challenges associated with using NGPs in clinical practice, the potential benefits and opportunities make it a promising area for further research and development.

**Table 10:** Comparison of Traditional and Next-Generation Probiotics

aspect	traditional probiotics	next-generation probiotics
discovery and development	discovered through personal experiences and historical use of fermented foods	identified through advanced techniques such as bioinformatics and next-generation sequencing (NGS) studies
safety	generally considered safe for human consumption with extensive historical use	safety needs thorough assessment due to novel strains and engineered modifications
mechanism of action	precise mode of action not fully understood, largely empirical	clear and targeted mechanisms of action based on genetic and metabolic engineering
microbial diversity	limited range of microorganisms, mostly from genera like *Lactobacillus* and *Bifidobacterium*	wide range of microbial genera and species, including engineered and novel strains
application scope	typically used to address general issues of suboptimal health and digestive support	designed to target specific diseases and conditions such as diabetes, obesity, cardiovascular diseases, and irritable bowel disease
formulation and delivery	mostly taken as dietary supplements in the form of capsules, powders, and fermented foods	potential to be used as biotherapeutics, possibly requiring specialized delivery systems
efficacy	variable efficacies, often dependent on the strain, dosage, and an individual’s microbiome	higher efficacy with targeted action, designed to interact with specific metabolic pathways and conditions
regulation	well-established regulatory guidelines with a history of safe use	emerging regulatory frameworks, with rigorous testing required for approval
personalization	limited ability to personalize, often a one-size-fits-all approach	high potential for personalization based on individual microbiome compositions and specific health needs
cost and production	generally lower cost with established production methods	potentially higher initial costs due to advanced R&D, but could decrease with scaled production and standardization

## 6 Understanding the Regulatory Categories of Probiotics

Regulatory aspects of probiotics encompass guidelines ensuring their safety, efficacy, and quality. Globally, these regulations vary but typically include stringent safety and quality standards, requiring scientific evidence for health claims, and precise labeling and marketing rules. In the United States, probiotics are regulated as dietary supplements by the Food and Drug Administration (FDA), while in the European Union, the European Food Safety Authority (EFSA) oversees their classification as food or novel foods. Japan categorizes them under Foods for Specialized Health Use (FOSHU) or Foods with Function Claims (FFC), and Canada regulates them as natural health products. Australia’s Therapeutic Goods Administration (TGA) treats probiotics as complementary medicines, and China requires a health food permit for those making health claims. India’s Food Safety and Standards Authority of India (FSSAI), Brazil’s Health Regulatory Agency (ANVISA), and regulatory bodies in Russia, South Korea, New Zealand, Switzerland, and Singapore also enforce specific safety and efficacy requirements (Table 11). These frameworks aim to ensure that probiotic products are safe, effective, and accurately marketed to consumers.^119^

**Table 11:** Regulatory Categories of Probiotics Across the Globe

region	regulatory category	description	ref
United States	dietary supplements	probiotics are regulated as dietary supplements under the *Dietary Supplement Health and Education Act* (DSHEA)	(219)
European Union	foods and food additives	probiotics can be regulated as food, food additives, or novel foods, depending on their use and claims	(220)
Japan	foods with health claims	probiotics are regulated as Foods for Specified Health Uses (FOSHU) or Foods with Function Claims (FFC)	(221)
Canada	natural health products	probiotics are regulated as natural health products (NHPs) under the Natural Health Products Regulations	(222)
Australia	complementary medicines	probiotics are regulated as complementary medicines by the Therapeutic Goods Administration (TGA)	(223)
China	functional foods	probiotics are regulated as functional foods with a health food permit required for products making health claims	(224)
India	dietary supplements	probiotics are regulated as dietary supplements or foods for special dietary uses under the Food Safety and Standards Authority of India (FSSAI)	(225)
Brazil	food supplements	probiotics are regulated as food supplements by the Brazilian Health Regulatory Agency (ANVISA)	(226)
Russia	food supplements	probiotics are regulated as dietary supplements and must comply with specific safety and efficacy requirements	(227)
South Korea	health functional foods	probiotics are regulated under the *Health Functional Food Act*, requiring safety and efficacy evidence	(228)
New Zealand	dietary supplements	probiotics are regulated as dietary supplements and foods, with specific guidelines for health claims	(229)
Switzerland	food and food additives	probiotics are regulated as food and must meet specific safety and efficacy standards for health claims	(230)
Singapore	health supplements	probiotics are regulated as health supplements with strict requirements for safety, efficacy, and labeling	(231)

## 7 Conclusions and Future Directions

Next-generation probiotics (NGPs) represent a transformative advancement in the field of microbiome-based therapies, offering a promising approach to preventing and treating chronic diseases such as diabetes, obesity, cardiovascular diseases, and inflammatory bowel disease (IBD). The current research underscores the potential of these advanced probiotics to provide more targeted, effective, and safer therapeutic interventions compared to traditional probiotics. By leveraging cutting-edge technologies such as synthetic biology, CRISPR/Cas9 gene editing, and gut microbiome engineering, researchers are developing probiotics that can modulate the gut microbiota with unprecedented precision, leading to significant improvements in clinical outcomes. The development of NGPs addresses several limitations associated with traditional probiotics, including their transient effects, lack of standardization, and variable efficacies among individuals. NGPs are designed to be more resilient, targeted, and tailored to individual microbiomic compositions, thereby enhancing their therapeutic potential. For instance, engineered strains, such as *Lactobacillus paracasei* producing conjugated linoleic acid (CLA) and *Akkermansia muciniphila* that improves glucose metabolism, exemplify the innovative approaches being taken to combat chronic diseases. Despite the promising results from preclinical studies and early phase clinical trials, the field of NGPs faces significant challenges, particularly in terms of regulatory approval and the need for extensive research to establish long-term safety and efficacy. Personalized approaches, although promising, require a deeper understanding of the complex interactions between probiotics and the host microbiome. Regulatory frameworks need to evolve to support the commercialization and clinical use of these advanced probiotics, ensuring they meet rigorous safety and efficacy standards.

The opportunities associated with NGPs are vast. They offer the potential for more specific and effective interventions with fewer side effects than traditional therapies, improving patient adherence and quality of life. Additionally, the use of NGPs could lead to cost savings for healthcare systems by reducing the need for expensive treatments and hospitalizations. The future of NGPs lies in continued research and development, focusing on understanding their mechanisms of action and optimizing their therapeutic potentials. As high-throughput sequencing and bioinformatics advance, new microbial strains with potential health benefits will be identified, further expanding the scope of NGPs. With rigorous clinical trials and regulatory support, NGPs could become a cornerstone of personalized medicine, offering targeted solutions for chronic diseases, and improving overall health outcomes.

Finally, NGPs hold great promise for advancing personalized medicine and enhancing the management of chronic diseases. Continued research and development, supported by evolving regulatory frameworks and technological advancements, are crucial to unlocking the full potential of NGPs. As our understanding of the gut microbiome deepens, NGPs are likely to play an increasingly important role in promoting health and treating a wide range of chronic conditions, marking a significant leap forward in the field of probiotic therapy.

## Data Availability

Data sharing is not applicable to this article, as no new data were created or analyzed in this study.
